# Cardiovascular Disease Burden: Evolving Knowledge of Risk Factors in Myocardial Infarction and Stroke through Population-Based Research and Perspectives in Global Prevention

**DOI:** 10.3389/fcvm.2015.00032

**Published:** 2015-08-13

**Authors:** Gustavo B. F. Oliveira, Alvaro Avezum, Leonardo Roever

**Affiliations:** ^1^Research Division, Dante Pazzanese Institute of Cardiology, São Paulo, Brazil; ^2^Clinical Research Center, Federal University of Uberlândia, Minas Gerais, Brazil

**Keywords:** cardiovascular diseases, burden of disease, risk factors, population-based research, global prevention

## Abstract

Current knowledge and research perspectives on the top ranking causes of mortality worldwide, i.e., ischemic heart disease and cerebrovascular diseases have developed rapidly. In fact, until recently, the evidence describing the incidence of acute myocardial infarction, the underlying risk factors, and the clinical outcomes of those who have this acute ischemic coronary event has largely been based on studies conducted in developed countries, with limited data for women and usually of low-ethnic diversity. Recent reports by the WHO have provided striking public health information, i.e., the global burden of cardiovascular mortality for the next decades is expected to predominantly occur among developing countries. Therefore, multiethnic population-based research including prospective cohorts and, when appropriate, case–control studies, is warranted. These studies should be specifically designed to ascertain key public health measures, such as geographic variations in non-communicable diseases, diagnosis of traditional and potential newly discovered risk factors, causes of death and disability, and gaps for improvement in healthcare prevention (both primary and secondary) and specific treatments. As an example, a multinational, multiethnic population-based cohort study is the Prospective Urban and Rural Epidemiology study, which is the largest global initiative of nearly 200,000 adults aged 35–70 years, looking at environmental, societal, and biological influences on obesity and chronic health conditions, such as ischemic heart disease, stroke, and cancer among urban and rural communities in low-, middle-, and high-income countries, with national, community, household, and individual-level data. Implementation of population-based strategies is crucial to optimizing limited health system resources while improving care and cardiovascular morbidity and mortality.

Globally, myocardial infarction is an important cause of premature death. Among its well-established modifiable risk factors are hypertension, dyslipidemia, smoking, and diabetes. However, the evidence describing the incidence of acute myocardial infarction, the underlying risk factors, and the clinical outcomes of those who have this acute ischemic coronary event has largely been based on studies conducted in developed countries, with limited data for women and usually of low-ethnic diversity. Therefore, generalizability to allow for public health implementation has not been properly feasible or has been made with high level of uncertainty (1).

We herein describe recent progress in the research-based knowledge through well-designed community-based cohorts and large case–control studies, widely conducted, i.e., including different geographic regions and diverse cultures, languages, income levels, and ethnicities. This research strategy obviously enhances external validity or broader generalizability of assessment of risk factors for acute myocardial infarction and stroke, thus enabling healthcare providers with reliable foundation to implement cost-effective prevention strategies on both community and population levels.

In order to address this paucity of precision and external validity, high-quality data from well-conducted studies over the last decade have provided cardiovascular field with clinically relevant information as well as additional insights on the risk factors associated with higher risk for the first myocardial infarction or stroke (2, 3). Those studies were designed to address the lack of representativeness of low-income countries and societal minorities as well as to evaluate the actual impact of traditional and emerging risk factors on the population attributable risk (PAR), i.e., variation in prevalence of a risk factor leads to different impact on specific population risk related to that particular factor.

The INTERHEART study was a multicenter, international, case–control study, designed to assess the importance of simple and easily identifiable risk factors for the occurrence of the first myocardial infarction worldwide (2). Between February/1999 and March/2003, 15,152 cases and 14,820 controls were enrolled in 262 centers from 52 countries in 7 major geographic regions of all continents. The most relevant finding was the determination of 9 risk factors that accounted for over 90% of the PAR of the first acute myocardial infarction. Moreover, those factors were easily measured and collected by standardized electronic data capture forms, and were considered potentially modifiable, which included the following by decreasing odds ratio: raised ApoB/ApoA1 ratio (3.25), current smoking status (2.87), psychosocial factors (2.67), diabetes (2.37), history of hypertension (1.91), abdominal obesity (1.12), alcohol consumption (0.91), regular physical activity (0.86), and daily consumption of fruits and vegetables (0.70), indeed all factors being significantly associated with acute myocardial infarction (*p* < 0.0001 for all risk factors and *p* = 0.03 for alcohol). Furthermore, the associations observed between these risk factors and acute myocardial infarction were consistent in both genders and across different ethnicities and geographic regions, enhancing the worldwide generalizability of the results.

Similarly, the INTERSTROKE pilot study (3) was also a large standardized case–control study in 22 countries globally, between March 1, 2007, and April 23, 2010, which was thought and conducted with the primary objective of evaluating the association of known as well as newly emerging risk factors with occurrence of the first stroke and its subtypes. In addition, the investigators aimed to assess individual contribution of specific risk factors to the stroke burden while exploring differences between risk factors for stroke and those for myocardial infarction. In the first 3,000 cases, 78% with ischemic stroke and 22% with intracerebral hemorrhagic stroke, and age- and gender-matched 3,000 controls, significant multivariable model-adjusted risk factors for all cases of stroke included history of hypertension, current smoking status relative to former or never, abdominal obesity measured by waist-to-hip ratio, ApoB/ApoA1 ratio, estimated unhealthy diet risk score, diabetes mellitus, alcohol intake, psychosocial stress and depression, lack of regular physical activity, and cardiac causes, which collectively accounted for nearly 90% (99% CI 82.3–92.2) of the PAR for strokes. These risk factors were significant for ischemic stroke, whereas smoking, alcohol intake, diet, hypertension, and waist-to-hip ratio were significantly associated with intracerebral hemorrhagic stroke.

Furthermore, the large INTERSTROKE study was recently presented at the World Congress of Cardiology 2014 Scientific Sessions, yet to be published (4). This second phase of the case–control study for stroke enrolled more than 27,011 individuals, including 13,604 with stroke and 13,407 age- and gender-matched controls. Patients were recruited from centers in North America; Western, Central, and Northern Europe; the Middle East; South America; China, South and Southeast Asia; and Africa. As expected, although still preliminary, the newly released results are concordant to those of the first phase of the INTERSTROKE abovementioned. The authors have identified a number of modifiable risk factors that explain approximately 90% of the PAR for stroke, with hypertension being the most important one. The other risk factors that contribute most to the stroke risk include lipid levels, physical inactivity, smoking, and diet. In addition, larger waist-to-hip ratios, a history of diabetes, increased alcohol intake, psychosocial stress and/or depression, and cardiac causes all contribute to risk for developing a first stroke.

Moreover, recent reports by the World Health Organization have provided striking public health information, i.e., the global burden of cardiovascular mortality for the next decades is expected to predominantly occur among developing countries. Current statistics of premature deaths due to cardiovascular diseases (CVD) ranges from 4% in high-income countries to astonishing estimate of 80% of the total CVD mortality occur in developing countries. This imbalance obviously leads to growing inequalities in the occurrence, outcomes, and heath care related costs of CVD across various income levels and populations (5).

Therefore, multiethnic population-based research including prospective cohorts and, when appropriate, case–control and cross-sectional studies, is warranted. Epidemiological and clinical studies should be specifically designed to ascertain key public health measures, such as geographic variations, in the prevalence and incidence of non-communicable diseases, diagnosis of traditional and potential newly discovered risk factors, causes of death and disability, and practice gaps. The pooled data will provide the rationale for proposals and implementation of feasible strategies, with primary goals of improvement in cardiovascular disease prevention, both primary – starting early in life or even during fetal development – and secondary, with sustained long-term accessibility, affordability, and patient adherence to available effective medications, and adoption of quality of care improvement programs.

Finally, current epidemiological research recognizes the continuous need for global, accurate, and reliable description of morbidity and mortality due to cardiovascular causes, which would enable comparisons while assessing the progress of delivery of benefit-proven cardiovascular care (6, 7).

As a good example of research with warranted broader geographic and ethnic representativeness, the multinational, population-based cohort Prospective Urban and Rural Epidemiology (PURE) study is the largest global initiative assessing societal, biological, and environmental influences on relevant chronic public health conditions, such as ischemic heart disease and stroke (8). The PURE study currently comprises nearly 200,000 adults aged 35–70 years who were initially enrolled between 2003 and 2009, from either urban or rural communities in low-, middle-, and high-income countries. Prespecified statistical assumptions were taken into consideration to collect huge amount of relevant data at national, community, household, and individual levels, since the baseline and throughout the long-term follow-up. Some of its recent contributions include the following: contemporary data on hypertension awareness, treatment, and control (9), i.e., in a large multinational population sample, 46.5% of participants with hypertension were aware of the diagnosis and blood pressure control was found in 32.5% of those receiving antihypertensive medications. Overall, those findings emphasize the substantial gap that requires improvement efforts by public health polices in terms of adequate hypertension diagnosis, broad accessibility to inexpensive treatments, and eventually targeted blood pressure control and lower morbidity and mortality.

In terms of non-pharmacological prevention strategies, among a sample of patients with prior coronary heart disease or cerebrovascular disease event from countries with various income levels, the proportion of individuals adopting all three healthy lifestyle behaviors, i.e., avoidance or complete smoking cessation, eating a healthy diet with regular, daily consumption of vegetables and fruits, and undertaking physical activities was only 4.3% (95% CI, 3.1–5.8%), with individuals from higher income countries being more likely to report a healthy behavior (10). Lastly, because sustained use of secondary prevention medications is not ideally offered worldwide – especially low in poorer countries and in rural-resident individuals – systematic, regionally customized feasible approaches are needed to improve the long-term use of simple, affordable and effective treatments, i.e., antiplatelets, betablockers, angiotensin-converting-enzyme inhibitors or angiotensin-receptor blockers, and statins in individuals with a history of coronary heart disease, stroke, or peripheral artery disease (11).

After taking this knowledge into consideration, what would be the next steps for prevention of myocardial infarction, stroke, heart failure, and related deaths? In order to achieve that goal of prevention while targeting significant reductions in morbidity and mortality, continuously growing body of evidence through population-based research and, consequently, worldwide implementation of simple and efficient large-scale primary and secondary prevention strategies are highly demanded and, therefore, should be a global priority on a multilevel organized task force.

Indeed, it is important to discuss how to apply newly developed evidence into daily practice, which certainly requires huge collaboration on global, political, governmental, societal, professional, and local levels to foster rapid, full, and measurable implementation of simple, widely available, cost-effective, and safe strategies of prevention and treatment targeting significant reductions in cardiovascular mortality. As recently proposed by the 25 × 25 World Heart Federation Program, the Global Cardiovascular Disease Taskforce calls on the CVD community to endorse, build local capacity with financial and human resources, and promptly make strong efforts on four key targets, i.e., fight against lack of regular physical activity, implementation of massive health polices for hypertension/blood pressure management, governmental policies for the reduction in dietary salt intake as well as tobacco taxation control and consumption cessation programs. By acting on a global perspective, we expect to achieve the feasible goal of 25% relative reduction in premature cardiovascular disease mortality by 2025 (12).

Furthermore, some recommendations have been recently released to provide guidance to improving cardiovascular health at the community level, which include some interventions for public health programs: broad surveillance, which is intended to guarantee that every person in all communities should have access to clear information about the public health impact of ischemic heart disease and cerebrovascular disease, i.e., the top ranking causes of death and disease-related disability and poor quality of life; general and specific health education and large-scale media; organizations and partnerships built at the community level; ensuring personal health services; environmental change and finally, policy change (13).

The WHF 25 × 25 Prevention Program expected risk reduction is in alignment with the WHO “Global Action Plan for the Prevention and Control of Non-Communicable Diseases 2013–2020,” which was endorsed by the World Health Assembly in May 2013. The WHO primary goal is a 25% relative reduction in the incidence of premature mortality due to non-communicable diseases, including CVD. It is relevant to mention some of the main principles of this global prevention plan: lifespan intervention approach, which makes sense in terms of prevention by avoidance of long exposure to risk factors as well as to the causes of the risk factors; organization, autonomy, continuing education and training, and required financial resources at the community and individual levels; rapid and sustained use of evidence-based strategies; universal health coverage; multiprofessional and multisectoral actions (14).

Therefore, healthcare providers, physicians, non-physicians, cardiovascular associations, and eventually governments/policy makers should adopt the current platform and perspectives, thought as being the scientific foundation, for continuous assessment of CVD burden as well as proposals and implementation of population-based strategies aimed at reducing morbidity, mortality, and healthcare costs, thus optimizing limited health system resources while improving care and clinical outcomes.

## Conflict of Interest Statement

The authors declare that the research was conducted in the absence of any commercial or financial relationships that could be construed as a potential conflict of interest.
